# Editorial: COVID-19 and hematological malignancies: Volume II

**DOI:** 10.3389/fonc.2022.1044582

**Published:** 2022-10-10

**Authors:** Lindsay Wilde, Adam Binder, Claudio Cerchione, Alessandro Isidori

**Affiliations:** ^1^ Department of Medical Oncology, Division of Hematologic Malignancy and Stem Cell Transplantation, Thomas Jefferson University Hospital, Philadelphia, PA, United States; ^2^ Hematology Unit, Istituto Scientifico Romagnolo per lo Studio e la Cura dei Tumori (IRST) IRCCS, Meldola, Italy; ^3^ Hematology and Stem Cell Transplant Center, Azienda Ospedaliera Ospedaliera Ospedali Riuniti Marche Nord (AORMN) Hospital, Pesaro, Italy

**Keywords:** COVID - 19, leukemia, lymphoma, myeloproliferative neoplasms, plasma cell dyscrasia

COVID-19 and Hematological Malignancies: Volume II is the second in a series of special Research Topics dedicated to the ongoing COVID-19 pandemic. The effects of COVID-19 on cancer care have been felt worldwide and touch all parts of the care continuum, including screening, diagnosis, treatment, and prognosis. Our understanding about the interplay between the virus and patients with hematologic malignancies is evolving and this group of articles serves to further that knowledge.

First in the series is a retrospective single-institution study evaluating the outcomes of 62 patients with hematologic malignancies who developed COVID-19 during the first year of the pandemic. Lymphoid malignancies were more common than myeloid malignancies in this cohort. The overall mortality rate of 16.1% was lower than some similar published studies, but increased to 28.6% in hospitalized patients and 60% in patients requiring ICU level care. Other factors, such as active treatment, history of tobacco use, and medical comorbidities did not seem to correlate with outcomes. Studies such as this provide important insight into COVID-19’s effect on patient outcomes, particularly in the early days of the pandemic before vaccines were available.

Next, two case reports of unique clinical scenarios in patients with lymphoma highlight the dilemmas that arise when COVID-19 affects this immunocompromised population. First, Capoluoungo et al. describe a patient with follicular lymphoma in remission after R-CHOP and undergoing maintenance rituximab therapy every two months who was diagnosed with COVID-19 in late August of 2020. She had a complicated 6-month hospital course throughout which she had persistently positive nasopharyngeal swab testing for COVID-19 and the absence of antibody development. Other studies have also noted this phenomenon in this patient population. Viral genomic analysis identified a previously unreported variant of SARS-CoV-2 with a mutation that may confer an ability to evade immune recognition. We now know that the emergence of SARS-CoV-2 variants can and will continue to have a significant impact on how the pandemic progresses. Second, Nilius-Eliwi et al. report a case of a patient with relapsed diffuse large B-cell lymphoma who developed COVID-19 with persistent nasopharyngeal swab positivity and was successfully treated with CAR-T cells (axicabtagene-ciloleucel) for progressive disease while actively infected. This case is emblematic of the difficult decisions that must be made when patients need urgent treatment for their malignancy during active COVID-19 infection and suggests that administration of CAR-T therapy may be safe for some patients.

Two studies in the series address the significant issue of COVID-19 vaccine response in patients with hematologic malignancies. Chiarucci et al. report their experience with administration of the mRNA-based BNT162b2 (Pfizer-BioNTech) vaccine in patients who underwent allogeneic or autologous stem cell transplant. They found that approximately 50% of allo-HSCT and 84% of auto-HSCT recipients were able to generate an antibody response after vaccination. Prior or concomitant rituximab or cyclosporine was associated with a poorer or absent response. Similarly, the retrospective study by Gung et al. evaluates mRNA-based vaccine (either Pfizer or Moderna) responses in patients with lymphoproliferative disorders or plasma cell dyscrasias. Highest response rates were seen in patients with multiple myeloma while patients with chronic lymphocytic leukemia had the lowest rates, as has been demonstrated in other studies. They also show that recent chemo or immunotherapy, particularly with rituximab or obinutuzumab based regimens, was associated with lower rates of response. These findings are important to consider when counseling patients about vaccine efficacy.

Finally, this series includes two survey studies that focus on the COVID-19 pandemic from the perspective of patients with hematologic malignancies. First, the prospective survey study by Cheikh et al. is an evaluation of the effectiveness of and patient satisfaction with safety measures that were put in place at Naef K. Basile Cancer Institute at the American University of Beirut Medical Center. This included modifications to clinic workflow, use of telemedicine, use of PPE, and public education. While the study involved patients with solid tumors and hematologic diseases (malignant and non-malignant), it provides insight into patient perceptions of compliance with safety measures at this institution. Second, the online survey of patients with polycythemia vera, essential thrombocytosis, and myelofibrosis conducted by Cavalca et al. and at the Myeloproliferative Disease Center of the Hematological Division of the S. Gerardo Hospital in Monza, Italy touches on the mental health impacts of the pandemic. While anxiety level was classified as normal for the majority of patients (78.8%), symptom burden worsened in 74.2% of patients, and patients who reported greater levels of anxiety reported marked worsening of symptoms. Studies of patient-reported outcomes, such as these, are and will continue to be vital in understanding the longer-term effects that COVID-19 has had on these vulnerable patients.

The Editors wish to thank all who contributed to this Research Topic, as well as all of the patients and members of the healthcare community who have worked together during this unprecedented time. As the pandemic progresses, more is being learned about how SARS-CoV-2 affects patients with hematologic malignancies and much research is ongoing. Volume III of this Research Topic series is in progress and we look forward to continuing to learn from the work that is being done worldwide.

## Author contributions

LW, AB, CC, and AI are co-editors of this Research Topic. The editorial was drafted by LW, and reviewed by AB, CC, and AI. All authors contributed to the article and approved the submitted version.

## Conflict of interest

The authors declare that the research was conducted in the absence of any commercial or financial relationships that could be construed as a potential conflict of interest.

## Publisher’s note

All claims expressed in this article are solely those of the authors and do not necessarily represent those of their affiliated organizations, or those of the publisher, the editors and the reviewers. Any product that may be evaluated in this article, or claim that may be made by its manufacturer, is not guaranteed or endorsed by the publisher.

